# Screen-Printed Wearable Sweat Sensor for Cost-Effective Assessment of Human Hydration Status through Potassium and Sodium Ion Detection

**DOI:** 10.3390/mi14081497

**Published:** 2023-07-26

**Authors:** Mingpeng Yang, Nan Sun, Xiaochen Lai, Yanjie Li, Xingqiang Zhao, Jiamin Wu, Wangping Zhou

**Affiliations:** 1 School of Automation, Nanjing University of Information Science and Technology, 219 Ningliu Road, Nanjing 210044, China; sunnan6047@163.com (N.S.); xchlai@nuist.edu.cn (X.L.); zxq8562@163.com (X.Z.); 2 Jiangsu Collaborative Innovation Centre on Atmospheric Environment and Equipment Technology, Nanjing University of Information Science and Technology, 219 Ningliu Road, Nanjing 210044, China; 3 Nanjing NARI Information and Communication Technology, Co., Ltd., 19 Chengxin Road, Nanjing 211106, China; wjm19920727@163.com

**Keywords:** wearable sweat sensor, screen-printing technology, hydration status, potassium ion detection, sodium ion detection

## Abstract

Human sweat is intricately linked to human health, and unraveling its secrets necessitates a substantial volume of experimental data. However, conventional sensors fabricated via complex processes such as photolithography offer high detection precision at the expense of prohibitive costs. In this study, we presented a cost-effective and high-performance wearable flexible sweat sensor for real-time monitoring of K^+^ and Na^+^ concentrations in human sweat, fabricated using screen printing technology. Initially, we evaluated the electrical and electrochemical stability of the screen-printed substrate electrodes, which demonstrated good consistency with a variation within 10% of the relative standard deviation (RSD), meeting the requirements for reliable detection of K^+^ and Na^+^ in human sweat. Subsequently, we employed an “ion-electron” transduction layer and an ion-selective membrane to construct the sensors for detecting K^+^ and Na^+^. Comprehensive tests were conducted to assess the sensors’ sensitivity, linearity, repeatability, resistance to interference, and mechanical deformation capabilities. Furthermore, we evaluated their long-term stability during continuous monitoring and storage. The test results confirmed that the sensor’s performance indicators, as mentioned above, met the requirements for analyzing human sweat. In a 10-day continuous and regular monitoring experiment involving volunteers wearing the sensors, a wealth of data revealed a close relationship between K^+^ and Na^+^ concentrations in human sweat and hydration status. Notably, we observed that consistent and regular physical exercise effectively enhanced the body’s resistance to dehydration. These findings provided a solid foundation for conducting extensive experiments and further exploring the intricate relationship between human sweat and overall health. Our research paved a practical and feasible path for future studies in this domain.

## 1. Introduction

Engaging in physical labor or undergoing physical training in hot environments results in excessive sweating, thereby increasing the risk of dehydration. Mild dehydration is characterized by symptoms such as thirst, reduced urine output, fatigue, and muscle cramps, while severe cases can lead to life-threatening heatstroke [1,2,3,4]. Unfortunately, tragic incidents of dehydration among workers in high-temperature environments occur with alarming frequency. Therefore, it is crucial to implement real-time monitoring of workers’ hydration status and effective alert systems to enhance their safety and overall well-being. Similarly, intensive physical training in extreme conditions is indispensable for cultivating highly skilled soldiers; however, it often leads to dehydration, physical exhaustion, and heatstroke [5,6,7,8]. Real-time monitoring of soldiers’ hydration status can effectively prevent these conditions and provide valuable data for adjusting and optimizing training programs, ensuring optimal performance while reducing the risk of injuries. Likewise, real-time monitoring of athletes’ hydration status plays a vital role in assessing their physical condition, facilitating the exploration of more effective training methods, and improving performance while minimizing the likelihood of injuries.

Various methods are available to assess human hydration status, including measurements of body weight changes, plasma osmolality, urine specific gravity, urine color, urine osmolality, and bioelectrical impedance [9,10,11,12]. However, these methods have limitations regarding their suitability for individuals engaged in high-temperature work or training, as well as their inability to provide continuous and real-time monitoring. In recent years, significant advancements have been made in the development of wearable sensors for real-time detection of human sweat, offering a potential solution to these limitations [13,14,15]. Human sweat contains a diverse range of solutes, including inorganic ions, organic molecules, amino acids, hormones, proteins, peptides, and other secretions [16,17]. Previous studies have investigated the correlation between sweat biomarkers and blood biomarkers, with a well-documented linear relationship between sweat glucose concentration and blood glucose concentration [18,19,20]. Furthermore, research focusing on human hydration status has revealed a close association between potassium and sodium ion concentrations in sweat and an individual’s hydration status [21,22].

However, research on the correlation between sweat biomarkers and human diseases is currently limited, and its development potential lags behind that of blood biomarkers. Further research on human sweat physiology and the translation of its findings into practical applications require substantial experimental results and validation. The advent of the big data era and the application of artificial intelligence methods hold promise for accelerating this research; however, the scarcity of sufficient data remains a significant challenge. Although several researchers have explored the feasibility of using sweat potassium and sodium ion concentrations to assess human hydration status, the existing methods primarily rely on photolithography for fabricating the necessary electrodes [23,24,25]. While these electrodes offer exceptional accuracy and detection performance, their high fabrication costs restrict their widespread utilization, hindering the collection of extensive medical data.

To overcome these limitations, some scholars have investigated the integration of sensor electrodes into materials such as clothing and paper [26,27,28,29]. However, the feasibility, consistency, and stability of such approaches are still in the early conceptual stages and require further investigation. In this context, the application of screen-printing technology for constructing ion-selective electrodes on flexible substrates emerges as an appealing solution for both research purposes and the large-scale production of sweat sensors to assess human hydration status [30,31,32,33,34]. Although the accuracy of screen-printed electrodes is slightly lower compared to photolithography, the cost-effectiveness and production efficiency are significantly superior. This opens up possibilities for the application of artificial intelligence in the field of sweat sensing.

In this study, we employed the screen-printing process to fabricate ion-selective electrodes on flexible substrates. We optimized the process parameters to enhance the electrode’s performance. Additionally, we conducted comprehensive evaluations of the electrodes’ detection capabilities, comparing them with expensive gold electrodes as the benchmark. Subsequently, we conducted real-time measurements of human hydration status using the fabricated electrodes to validate the feasibility of the proposed method. Due to the inexpensive screen-printing fabrication technique, we were able to easily obtain a large number of electrodes with outstanding performance. We conducted a significant amount of testing and collected a wealth of sweat detection data, further confirming the correlation between potassium and sodium ion concentrations in sweat and the hydration status of the human body. Through continuous monitoring data over a period of 10 days, it was concluded that maintaining a regular exercise routine can effectively enhance the body’s resistance to dehydration.

## 2. Experiment

### 2.1. Materials, Reagents, and Equipment

Polyethylene terephthalate (PET) films were obtained from Shenzhen Taiyuan Plastic Materials Co., Ltd. Ag/AgCl ink was obtained from Shanghai Julong Electronic Technology Co., Ltd. (Shanghai, China). Carbon (CH-8) and insulator inks (IN-15M) were purchased from Jujo Chemical Co., Ltd. (Tokyo, Japan). The silk screen printing was commissioned to Deyun Printing Equipment Co., Ltd. (Changsha, China) for preparation.

All chemicals were used as received. 4-tert-Butylcalix[4]arene-tetraacetic acid tetraethyl ester (>96.0%) was purchased from Beijing Bailingwei Technology Co., Ltd. (Beijing, China). Bis(2-ethylhexyl) sebacate (DOS) (97%), valinomycin (≥95%), and poly (sodium 4-vinyl benzenesulfonate) (NaPSS) were purchased from Shanghai Yien Chemical Technology Co., Ltd. (Shanghai, China). Sodium tetraphenylborate (NaTPB) (99.5%), sodium tetrakis (3,5-bis(trifluoromethyl) phenyl) borate (Na-TFPB) (≥98.0%), cyclohexanone (99.8%), polyvinyl butyral (PVB), and polyvinyl chloride (PVC) were purchased from Aladdin Bio-Chem Technology Co., Ltd. (Shanghai, China). Tetrahydrofuran (THF) (99.5%) and potassium ferrocyanide (K_3_[Fe(CN_6_)]) (98%) were purchased from Shanghai Macklin Biochemical Co., Ltd. (Shanghai, China). 2,3-dihydrothieno[3,4-b] [1,4] dioxine (EDOT) (99%) was purchased from Shanghai Bide Pharmaceutical Technology Co., Ltd. (Shanghai, China). Methanol (≥99.7%), ethanol (≥99.7%), sodium chloride (NaCl) (≥99.8%), potassium chloride (KCl) (≥99.8%), magnesium chloride hexahydrate (MgCl_2_·6H_2_O) (98%), and calcium chloride (CaCl_2_) (96%) were purchased from Sinopharm Chemical Reagent Co., Ltd. (Shanghai, China). Deionized ultra-pure water (18 MΩ·cm) was used throughout.

The silk printing machine was purchased from Zhejiang Huaqi Zhengbang Automation Technology Co., Ltd. (Lishui, Zhejiang, China). The drying oven was purchased from Dongguan Lixian Instrument Technology Co., Ltd. (Dongguan, Guangdong, China). The electrochemical workstation was purchased from Shanghai Chenhua Instrument Co., Ltd. (Shanghai, China). Ag/AgCl electrodes were purchased from Shanghai Chenhua Instrument Co., Ltd. (Shanghai, China) and were used as reference electrodes in solid-state ion production.

The laboratory maintains precise control over temperature, maintaining it within the range of 22 °C to 24 °C, and ensures a relative humidity level of 55% to 60%. It is worth noting that, unless explicitly stated otherwise, all the experiments were carried out in the controlled laboratory environment mentioned above. By maintaining a controlled temperature and relative humidity, we aim to minimize the influence of environmental factors on our measurements and ensure the accuracy and validity of our findings.

### 2.2. Design and Fabrication of the Sensors

#### 2.2.1. Overall Design

**Figure 1:**
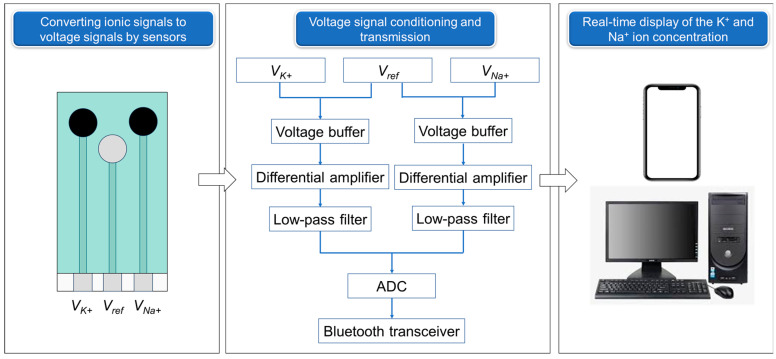
Overall scheme of human hydration status assessment.

The overall scheme of the human hydration status assessment is illustrated in Figure 1. Firstly, the screen-printing process was employed to fabricate the substrate electrodes and insulating layer on PET films. Subsequently, K^+^ and Na^+^ selective membranes, along with an Ag/AgCl reference electrode, were constructed on the substrate electrodes, resulting in the creation of K^+^ and Na^+^ sensors. During the detection process, a potential difference was generated between the K^+^ electrode and the Ag/AgCl reference electrode, as well as between the Na^+^ electrode and the Ag/AgCl reference electrode. The voltage signal was then passed through a signal conditioning and transmission module, which performed operations such as differential amplifying, filtering, and conversion into digital signals. The processed signal was transmitted to a mobile or computer platform using a Bluetooth module, enabling real-time visualization of changes in K^+^ and Na^+^ concentrations. This facilitated the assessment of an individual’s hydration status based on the detected variations.

#### 2.2.2. Structure of the Sensor

**Figure 2:**
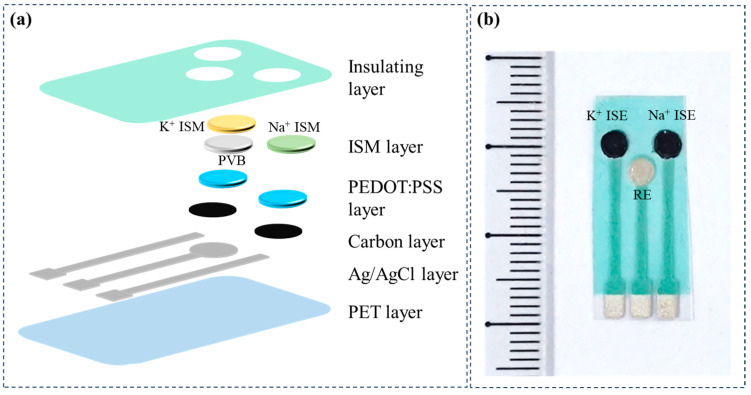
(**a**) Structure and (**b**) actual photograph of the sensor.

As shown in Figure 2a, the sensor is comprised of six layers: the PET layer, the Ag/AgCl layer, the carbon layer, the PEDOT: PSS layer, the ISM layer, and the insulating layer. The first three layers (PET layer, Ag/AgCl layer, and carbon layer) form the substrate electrodes responsible for facilitating the conduction of electrical signals. The PEDOT: PSS layer serves as the transducing layer, converting ion signals into electrical signals [35,36,37]. The ISM layer functions as an ion-selective membrane, enabling the passage of target ions while preventing interference from other ions. Simultaneously, a PVB solution is applied to coat the Ag/AgCl electrode, establishing a reference electrode. The insulating layer is crucial for safeguarding the electrical signal layer against short circuits and providing insulation. The actual photograph of the sensor is shown in Figure 2b.

#### 2.2.3. Fabrication of the Substrate Electrodes

The electrode patterns were meticulously designed using the graphic design software CorelDRAW for precise visualization. Each sensor consisted of one reference electrode and two working electrodes, all of which were circular with a diameter of 3 mm. The screen-printing technique, employing a 200-mesh screen, was utilized to create the desired electrode patterns, as depicted in Figure S1.

To accommodate the dimensions of the screen-printing patterns, PET films with a thickness of 100 μm were carefully cut, ensuring a size slightly larger than the screen dimensions of 60 × 100 mm, resulting in final dimensions of 80 × 120 mm. The cut PET films then underwent a thorough cleaning process, starting with immersion in anhydrous ethanol for 5 min, followed by ultrasonic cleaning. Afterward, the films were allowed to air dry. Subsequently, they were subjected to an additional 5 min of ultrasonic cleaning using deionized water and then dried in a 90 °C oven.

During the screen-printing process, the distance between the screen plate and the base was precisely set at 2 mm, while the blade angle was adjusted to 75 degrees. The screen plate, as shown in Figure S1a, was securely positioned above the base, and the Ag/AgCl ink was poured onto the plate to initiate the printing of the first layer while simultaneously moving the blade to create the Ag/AgCl coating. This layer was effectively cured by subjecting it to a baking process at 90 °C for 15 min. Following a similar procedure, the second layer (Figure S1b), consisting of conductive ink, was carefully printed and subsequently cured under identical conditions. Finally, the insulating layer (Figure S1c) was printed using insulating ink, which was then cured by exposing it to a UV curing lamp (175 W) for 1 min. To ensure optimal performance, the screen plate and blade were thoroughly cleaned using screen cleaning water after each printing session.

Once the screen-printing process was completed, the PET films were meticulously cut into individual electrodes (Figure S1d). These electrodes were then securely sealed within plastic bags and stored in a dry and dark cabinet, ensuring their preservation for future use.

#### 2.2.4. Preparation of the Ion Selective Membranes and the Reference Electrode

The sodium ion-selective membrane cocktail was prepared following these steps. A solution of 660 μL tetrahydrofuran was used, into which 1 mg of 4-tert-butylcalix[4]arene-tetraacetic acid tetraethyl ester, 0.55 mg of sodium tetrakis(3,5-bis(trifluoromethyl)phenyl)borate (Na-TFPB), and 65.45 mg of di-n-octyl sebacate (DOS) were added. The mixture was stirred with a magnetic stirrer for 15 min and sonicated for 15 min to ensure complete dissolution, resulting in a clear and transparent solution. Next, 33 mg of polyvinyl chloride (PVC) was introduced into the solution, and the stirring and sonication processes were repeated for 15 min until complete dissolution was achieved. The sodium ion-selective solution was then sealed and stored at 4 °C for further use.

Similarly, the potassium ion-selective membrane cocktail was prepared using the following procedure: A solution of cyclohexanone was utilized, and 2 mg of valinomycin, 0.5 mg of NaTPB, and 64.7 mg of DOS were added to it. The mixture was stirred with a magnetic stirrer for 15 min and sonicated for 15 min to dissolve the components. Subsequently, 32.7 mg of PVC was added to the solution, and stirring and sonication were continued for 15 min until complete dissolution was achieved. The potassium ion-selective solution was then sealed and stored at 4 °C.

To prepare the PVB solution, 79.1 mg of PVB powder and 50 mg of sodium chloride were added to 1 mL of methanol. The mixture was thoroughly stirred for 2 min and sonicated for 30 min to ensure complete dissolution. The reference solution was then sealed and stored at 4 °C.

The “ion-electron” transducing layer of the PEDOT: PSS film was prepared using the following method: Initially, solid-state electrodes were subjected to ultrasonic cleaning for 5 min using anhydrous ethanol, followed by deionized water, to remove any residual impurities from the electrode surface. The cleaned electrodes were then immersed in an electroplating solution consisting of 0.01 M EDOT and 0.1 M NaPSS [38,39]. Electroplating was performed using a three-electrode electrochemical system with chronoamperometry. A constant current of 14 μA was applied with a sampling interval of 5 ms, resulting in a total electroplating time of 714 s. After the electroplating process, the electrodes were rinsed with deionized water and left to dry overnight.

For the preparation of the potassium and sodium ion-selective membranes, the following steps were followed: A microsyringe was used to coat the reference electrode with a 0.5 μL solution of PVB, applying four drops in total. Each 0.5 μL application required a drying time of 20 min, and the electrode was left to air-dry overnight at room temperature to ensure complete solvent evaporation. Similarly, the corresponding selective electrodes were coated with a 0.5 μL solution of ion-selective material using a microsyringe, applying four drops in total. Each 0.5 μL application required a drying time of 20 min, and the electrode was left to air-dry overnight at room temperature to ensure complete solvent evaporation.

#### 2.2.5. Signal Treatment

Sodium ions (Na^+^) and potassium ions (K^+^) traverse the ion-selective membrane and, through the “ion-electron” transduction layer of the PEDOT: PSS film, generate an output potential signal. This signal is then differentially compared with the reference potential signal from the Ag/AgCl electrode. The resulting voltage signal demonstrates a linear correlation with the ion concentration. However, direct reading of this signal by the upper computer is not possible. To address this, the hardware circuitry of the sensor has been meticulously designed in this study. It entails buffering the raw electrical signal, performing differential amplification, applying low-pass filtering, and performing analog-to-digital conversion (ADC). Subsequently, the signal is transmitted to the terminal upper device (such as a smartphone or computer) via a Bluetooth module, enabling the final signal reading. The circuit conditioning diagram and the physical representation of the flexible printed circuit board (FPBC) are depicted in Figure S2. Since the conditioning circuit for potassium ions is identical to that for sodium ions, only the potassium ion circuit is illustrated in this context.

### 2.3. Performance of the Substrate Electrodes

The screen-printing process is widely acknowledged for its cost-effectiveness. However, when compared to electrodes fabricated using photolithography, screen-printed electrodes may exhibit relatively weaker repeatability in terms of their electrical performance. Therefore, this study conducted an initial assessment of their conductivity. Specifically, five substrate electrodes were randomly selected from the same batch, and the resistance values of the two working electrodes and the reference electrode were measured for each of them. Furthermore, another set of five substrate electrodes, sourced from different batches, were randomly chosen, and the respective resistance values of the two working electrodes and the reference electrode were also measured. Subsequently, the average resistance value and the relative standard deviation were computed for each measurement.

The electrochemical performance of the screen-printed electrodes was assessed through cyclic voltammetry (CV). The CV measurements were conducted by separately scanning between the two working electrodes and the Ag/AgCl reference electrode on the substrate electrodes. These measurements took place in a 2 mM K_3_[Fe(CN_6_)] solution with 0.1 M KCl. The scanning was performed at a rate of 0.1 V/s, covering a voltage range from −0.4 V to 0.9 V. To evaluate the repeatability of the electrochemical measurements, a random substrate electrode was selected, and each of its two working electrodes was subjected to five consecutive scanning cycles. This assessment aimed to determine the within-electrode repeatability of electrochemical detection. Additionally, from the same batch, five random substrate electrodes were chosen, and each substrate electrode’s two working electrodes underwent a single cycle of scanning. This analysis aimed to evaluate the repeatability of the electrochemical measurements within the same batch of substrate electrodes. Furthermore, from a pool of five batches, five random substrate electrodes were selected, and each substrate electrode’s two working electrodes were scanned for one cycle. This evaluation aimed to assess the repeatability of the electrochemical measurements across different batches of substrate electrodes. The relative standard deviation (RSD) of the peak current values obtained from the CV curves was calculated to evaluate repeatability within the same batch and across different batches of substrate electrodes.

### 2.4. Detection Performance of the Sensors

The potentiometric measurement method was employed to detect the concentrations of K^+^ and Na^+^ ions in sweat. An electrochemical workstation was utilized to conduct the open-circuit potential method. The sensor’s reference electrode consisted of a PVB-modified Ag/AgCl electrode, while the working electrodes were the K^+^ and Na^+^ ion-selective electrodes. The exposed sensing end of the sensor, including the K^+^, Na^+^, and reference electrodes, was immersed in the test solution. The open-circuit potential (OCP) value was recorded for a duration of 60 s. The aforementioned detection method was consistently utilized for all experimental evaluations pertaining to the assessment of the sensor’s performance.

#### 2.4.1. Sensitivity and Linearity

The sensitivity and linearity of the sensor were evaluated considering the concentration ranges of potassium and sodium ions in human sweat, which are approximately 1–24 mM [40,41,42] and 8–128 mM [22,43,44], respectively. Consequently, the testing range of the sensor was determined based on these concentration ranges. The sensing end of the sensor was immersed in NaCl solutions of various concentrations (5 mM, 10 mM, 20 mM, 40 mM, 80 mM, and 160 mM) as well as KCl solutions with different concentrations (1 mM, 2 mM, 4 mM, 8 mM, 16 mM, and 32 mM). Each immersion lasted for 30 s, during which the open circuit potential (OCP) was recorded. Three replicates were performed at each concentration, and the average value was calculated. The obtained data were subjected to linear regression using the least squares method to determine the sensor’s sensitivity and linearity. Calibration curve measurements for the K^+^ ISE and Na^+^ ISE were conducted, spanning an extended concentration range (from 10^−7^ to 10^−1^ mol L^−1^). This systematic approach allowed us to determine the upper and lower detection limits of the sensor, aiming to achieve a thorough understanding of its analytical capabilities.

#### 2.4.2. Repeatability

To assess the run-to-run repeatability of the sensor’s detection performance, calibration curve tests were conducted five times for potassium ions (1 mM, 2 mM, 4 mM, 8 mM, 16 mM, and 32 mM) and sodium ions (5 mM, 10 mM, 20 mM, 40 mM, 80 mM, and 160 mM) using the same sensor. Each test was conducted with an interval of 1 h for the reconditioning of the ISEs. Additionally, the chip-to-chip repeatability of the sensor’s detection performance was evaluated by performing calibration curve tests for potassium and sodium ions using five different sensors. During each calibration curve measurement, the tests followed a sequential process, beginning with solutions at lower concentrations and gradually progressing to higher concentrations. Within each test interval, the chip underwent a brief wash with ultra-pure water, lasting 5–10 s. Additionally, between different calibration curves, the chip was thoroughly rinsed with ultra-pure water.

#### 2.4.3. Interference Resistance

Other ions present in human sweat may interfere with the detection of target ions, making it necessary to evaluate the sensor’s interference resistance. The sensor was immersed in the test ion solution, and interfering ions were sequentially added while the solution was stirred using a magnetic stirrer. The voltage output detected by the sensor was observed for any changes. For interference resistance testing regarding Na^+^, the sensor was first immersed in a 50 mL solution of 10 mM NaCl. Once the output voltage signal stabilized, recording began. Then, 5 mM MgCl_2_, 5 mM CaCl_2_, and 5 mM KCl were sequentially added. Finally, a high concentration of NaCl solution was added to achieve a NaCl concentration of 20 mM in the solution.

For interference resistance testing regarding K^+^, the sensor was initially immersed in a 50 mL solution of 2 mM KCl. Once the output voltage signal stabilized, recording commenced. Subsequently, 5 mM MgCl_2_, 5 mM CaCl_2_, and 5 mM NaCl were sequentially added. Finally, a high concentration of KCl was added to achieve a KCl concentration of 4 mM in the solution.

#### 2.4.4. Mechanical Deformation Resistance

When wearable sensors are in use, they are in direct contact with the human skin and may undergo mechanical deformation due to body movements. This mechanical deformation can potentially cause instability in sensor readings. Therefore, it is necessary to test the sensor’s resistance to mechanical deformation. The sensor’s output voltage was measured when subjected to bending deformations of 0°, 180°, and −180°, while immersed in a 2 mM KCl solution and a 10 mM NaCl solution, respectively.

#### 2.4.5. Stability

The developed sensors in this paper are intended for the detection of K^+^ and Na^+^ ion concentrations in human sweat, allowing for the evaluation of the individual’s hydration status. As the detection process typically requires a considerable amount of time after the sensors’ calibration, it is essential to assess the stability of the sensor data throughout the detection period. To accomplish this, the sensors were immersed individually in a 50 mL solution of 2 mM KCl and a 50 mL solution of 10 mM NaCl. Based on the measurement results presented in Figure S3, a settling time of one minute was allocated to allow the data to stabilize. Subsequently, voltage values were recorded over a 40-minute testing duration.

#### 2.4.6. Storage Stability

The sensor undergoes calibration curve testing every week for a duration of three weeks to observe the changes in sensitivity and baseline over time. After each sensor test, thorough rinsing with deionized water is conducted, followed by drying at room temperature (22–24 °C) for 12 h. Subsequently, the sensors are stored in a light-free and dry environment at room temperature (22–24 °C).

### 2.5. On-Body Trials

To verify the practical performance of the sensor, a real-world test using human sweat was conducted. During the test, the sensor was securely affixed to the volunteer’s arm. The acquired signals were modulated and processed using the prepared flexible printed circuit board (FPCB) and transmitted to a laptop for real-time data display and storage.

The developed sensor was utilized for a 10-day sweat test on the volunteer. Throughout the testing period, the participants maintained their regular diet and hydration. The testing sessions were conducted daily from 18:00 to 18:40. One hour prior to each test, the volunteer consumed 300 mL of tap water that had been boiled and cooled down to room temperature (22–24 °C). No further food or water intake was permitted during the testing period. During the test, the volunteer engaged in a 40-minute running exercise on a treadmill at a constant speed of 10 km/h and a 0-degree incline in a gymnasium. The ambient temperature in the gymnasium was maintained between 22 and 24 °C, with a relative humidity of 55% to 60%. Before conducting the test, the sensor underwent calibration using standard solutions and was rinsed with ultra-pure water. The volunteer’s skin in the area where the wearable device was placed was carefully wiped with medical alcohol and thoroughly washed with ultra-pure water. Subsequently, the sensor continuously measured the concentrations of K^+^ and Na^+^ in the volunteer’s sweat. A comprehensive measurement on the human body entails a 75-minute process, comprising 30 min for sensor calibration and cleaning, 5 min for skin preparation, and 40 min for the actual device measurement.

Additionally, the volunteer’s body weight was measured before and after each exercise session, and the changes in weight were recorded for comparison.

## 3. Results and Discussions

### 3.1. Electrical and Electrochemical Performance of Screen-Printed Substrate Electrodes

#### 3.1.1. Electrical Performance

The conductivity performance of substrate electrodes of sensors, both from the same batch and different batches, was examined. The results are summarized in Table 1. It is evident that the Ag/AgCl reference electrode exhibits a resistance of approximately 1.2 Ω, whereas the substrate electrodes for K^+^ and Na^+^ ISE demonstrate a resistance of approximately 76.5 Ω. The noticeable increase in resistance is attributed to the electrochemical stability requirements of the working electrode, necessitating the coating of a carbon layer on the substrate electrode. By comparing the standard deviation results, it becomes apparent that the electrode resistance consistency is superior among sensors from the same batch as opposed to different batches. Moreover, the standard deviation is relatively small in relation to the average electrode resistance, thus indicating excellent conductivity of the electrodes fabricated through screen printing along with remarkable consistency.

#### 3.1.2. Electrochemical Performance

**Figure 3:**
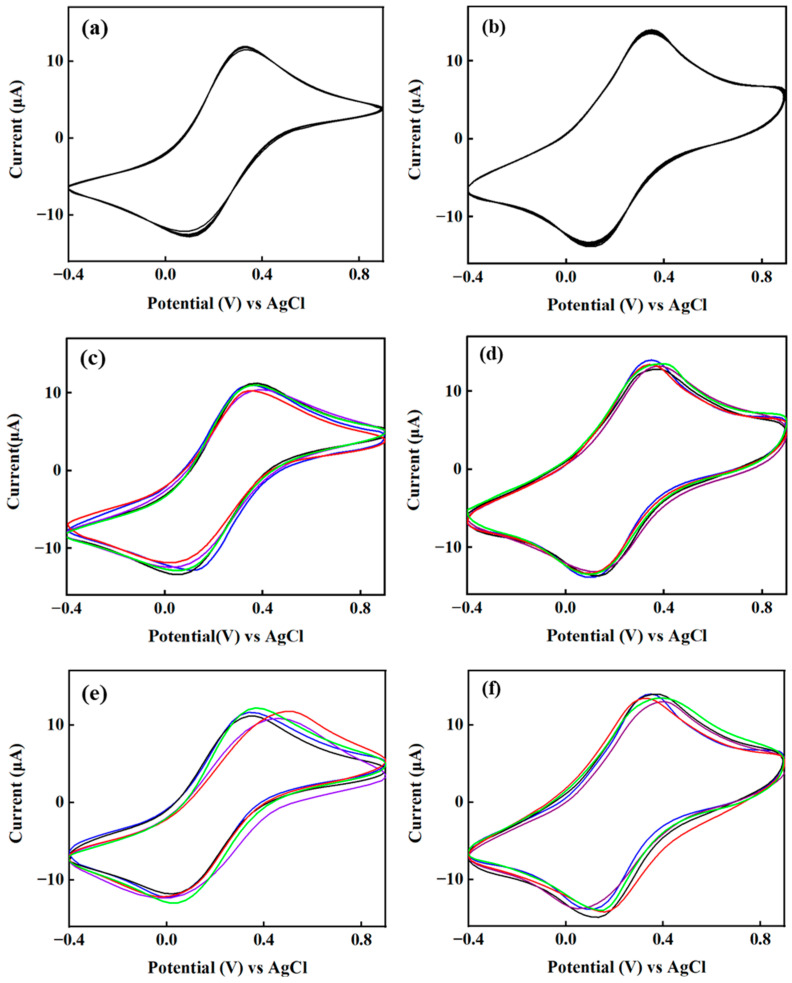
CV curves of the sensor’s substrate electrodes in a 2 mM K_3_[Fe(CN_6_)] solution containing 0.1 M KCl. (**a**) CV curves of a single K^+^ substrate electrode over 5 cycles; (**b**) CV curves of a single Na^+^ substrate electrode over 5 cycles; (**c**) CV curves of 5 different K^+^ substrate electrodes from the same batch over 5 cycles; (**d**) CV curves of 5 Na^+^ substrate electrodes from the same batch over 5 cycles; (**e**) CV curves of 5 different K^+^ substrate electrodes from different batches over 5 cycles; (**f**) CV curves of 5 Na^+^ substrate electrodes from different batches over 5 cycles. Note: K^+^ ISE, Na^+^ substrate electrodes, and the Ag/AgCl RE tested here are all the substrate electrodes, without any ISM or PVB coatings. The lines with the same color in sub-figures (**a**,**b**) represent CV results obtained from the same electrode, while the lines with different colors in sub-figures (**c**–**f**) represent CV results obtained from different electrodes.

The electrochemical stability and consistency of the sensor’s electrodes are crucial indicators of electrode performance. Figure 3 showcases the electrochemical properties of electrodes fabricated using the screen-printing technique. Figure 3a,b displays the cyclic voltammetry (CV) curves of the K^+^ and Na^+^ substrate electrodes, respectively, from the same sensor in a 2 mM K_3_[Fe(CN_6_)] solution with 0.1 M KCl over 5 cycles. It is evident that the CV curves from the 5 cycles exhibit significant overlap, indicating exceptional electrochemical stability of the electrodes.

Figure 3c,d demonstrates the CV curves of the K^+^ and Na^+^ substrate electrodes, respectively, from five different sensors within the same batch, with relative standard deviations (RSD) of 3.93% and 3.82%, respectively. On the other hand, Figure 3e,f depicts the CV curves of the K^+^ and Na^+^ substrate electrodes from 5 sensors across different batches, exhibiting RSD values of 9.73% and 8.86%, respectively. These findings suggest that the deviations in the CV curves among different electrodes are well controlled within 10%, falling within an acceptable range, thereby indicating excellent consistency.

### 3.2. Detection Performance of the Sensor

In order to evaluate the detection performance of the screen-printed wearable sensor, various aspects, including calibration performance, repeatability, interference resistance, stability, and storage life, were systematically assessed.

#### 3.2.1. Sensitivity and Linearity

**Figure 4:**
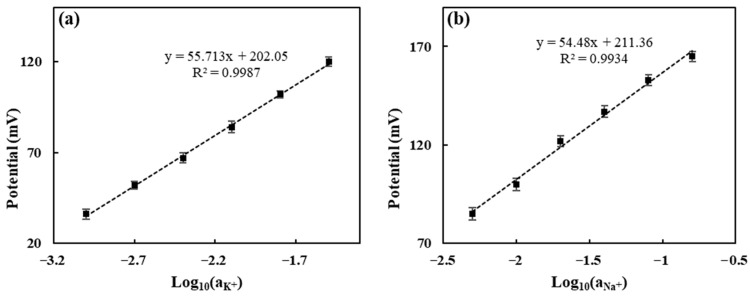
Calibration curves of the K^+^ ISE (**a**) and Na^+^ ISE (**b**) of the sensor (*n* = 3).

The calibration curves for the K^+^ ISE and Na^+^ ISE were determined. For the K^+^ ISE, calibration tests were conducted at specific concentrations (1 mM, 2 mM, 4 mM, 8 mM, 16 mM, and 32 mM). Three measurements were taken at each concentration, and the average values were obtained. Similarly, for the Na^+^ ISE, calibration tests were performed at designated concentrations (5 mM, 10 mM, 20 mM, 40 mM, 80 mM, and 160 mM), with three measurements taken at each concentration and averaged. The logarithm of the concentration was plotted on the *x*-axis, while the measured open-circuit potential was represented on the *y*-axis, resulting in the calibration curve (Figure 4). The calibration curve of the K^+^ ISE (Figure 4a) exhibited a slope of 55.731 mV/decade, while the calibration curve of the Na^+^ ISE (Figure 4b) exhibited a slope of 54.480 mV/decade. These values closely aligned with the theoretical value of 59.2 mV/decade as determined by the Nernst equation, falling within an acceptable range. Both calibration curves demonstrated a coefficient of determination (R^2^) greater than 0.99, indicating excellent linearity within their respective measurement ranges.

The calibration curves of the two ISEs of the sensor were obtained over an extended concentration range (from 10^−7^ to 10^−1^ mol L^−1^), as depicted in Figure S4 (Supporting Information). For the K^+^ ISE, two distinct slopes were observed within this range: the lower concentration range (from 10^−7^ to 10^−5^ mol L^−1^) exhibited a sub-Nernstian response with a lower slope value, while the higher concentration range (from 10^−5^ to 10^−1^ mol L^−1^) demonstrated a near-Nernstian slope. Similar trends were observed for the Na^+^ ISE. Consequently, the lower detection limits for the K^+^ and Na^+^ ISE were determined to be 10^−5^ mol L^−1^ and 10^−4^ mol L^−1^, respectively, within the near-Nernstian response range (the detection limits could be much lower for the sub-Nernstian response range, but the sensitivities were lower). These detection limits are below the concentration limits of K^+^ and Na^+^ in human blood. Moreover, both ISEs exhibited an upper limit exceeding 10^−1^ mol L^−1^, far surpassing the upper concentrations of K^+^ and Na^+^ in human sweat.

#### 3.2.2. Repeatability

**Figure 5:**
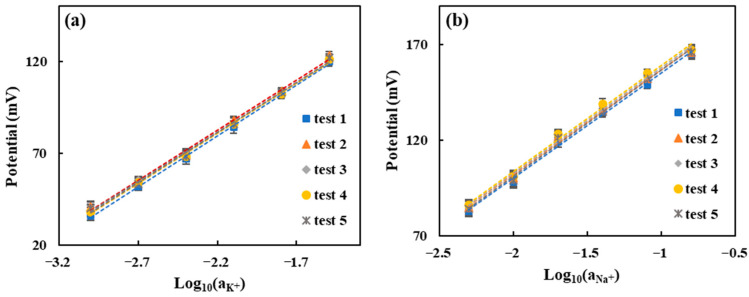
Repeatability testing performance of the same sensor’s K^+^ ISE (**a**) and Na^+^ ISE (**b**) (*n* = 5).

To evaluate the detection repeatability of the sensor, the study conducted tests on its two ion-selective electrodes. Specifically, the repeatability of the same sensor was assessed first, as illustrated in Figure 5. The results revealed that the calibration curves of the K^+^ ISE and Na^+^ ISE of the sensor exhibited a high degree of overlap, as shown in Table 2. Both the sensitivity (slope of the calibration curve) and the base potential remained stable, with consistently low standard deviation values across multiple measurements, indicating excellent performance.

**Figure 6:**
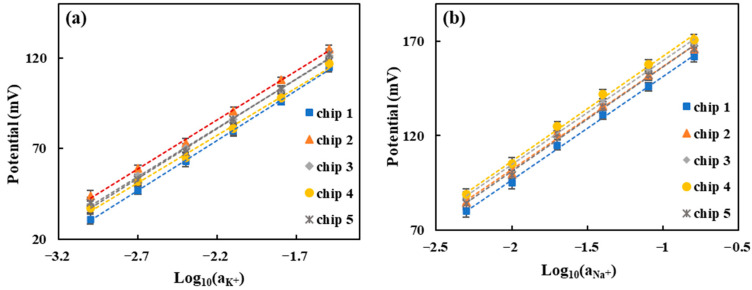
Repeatability testing performance of different sensors’ K^+^ ISE (**a**) and Na^+^ ISE (**b**) (*n* = 5).

Regarding the repeatability testing of different sensors, the results are presented in Figure 6. The findings demonstrated that the calibration curves of the K^+^ ISE and Na^+^ ISE for different sensors displayed slight deviations compared to the results of the same sensor. The calibration curves exhibited some vertical shifts, and the standard deviation of the base potential was slightly larger than that of the same electrode. However, these variations remained within an acceptable range. Additionally, the standard deviation of the sensitivity was marginally higher than that of the same electrode. Nonetheless, the overall repeatability indicators remained excellent.

In summary, the hydration status sensors fabricated using the screen-printing technique exhibited remarkable detection repeatability.

#### 3.2.3. Interference Resistance

Due to the presence of various interferents in human sweat, apart from the target analytes, the detection results may be influenced. Therefore, this study aimed to evaluate the sensor’s ability to resist interference from other substances. The performance of the potassium and sodium ISE, fabricated using screen printing techniques, was investigated.

**Figure 7:**
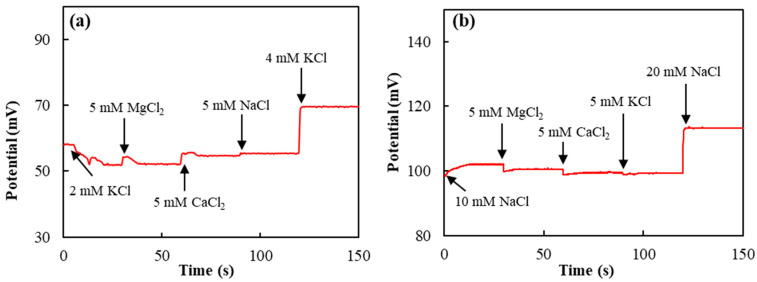
Test results regarding the sensor’s ability to resist interference from other ions. (**a**) Voltage responses of the K^+^ ISE were measured as 5 mM MgCl_2_, 5 mM CaCl_2_, 5 mM NaCl, and 4 mM KCl and sequentially added to a 2 mM KCl solution; (**b**) voltage responses of the Na^+^ ISE were recorded as 5 mM MgCl_2_, 5 mM CaCl_2_, 5 mM KCl, and 20 mM NaCl and successively introduced into a 10 mM NaCl solution.

For the K^+^ ISE of the sensor, the addition of 5 mM MgCl_2_, 5 mM CaCl_2_, and 5 mM NaCl to the test solution did not cause significant fluctuations in the voltage signal, with fluctuations within 3 mV (Figure 7a), which is within an acceptable range. However, when KCl solution was added to the solution, increasing the K^+^ concentration from 2 mM to 4 mM, the voltage signal increased by 16.5 mV, indicating a noticeable jump. Similarly, for the Na^+^ ISE, the sequential addition of 5 mM MgCl_2_, 5 mM CaCl_2_, and 5 mM KCl to the reaction chamber did not cause significant fluctuations in the voltage signal, with fluctuations within 2 mV (Figure 7b), indicating a minimal impact. However, when NaCl solution was added to the solution, increasing the Na^+^ concentration from 10 mM to 20 mM, the voltage signal increased by 16.8 mV, showing a clear jump.

These results demonstrate that the potassium and sodium ISE prepared using screen printing techniques exhibit excellent resistance to interference from other ions, displaying good ion selectivity. This characteristic makes it well-suited for practical applications in measuring human sweat. The observed ripples in the test curves can be attributed to disturbances caused by the magnetic stirrer during the testing process.

#### 3.2.4. Mechanical Deformation Resistance

**Figure 8:**
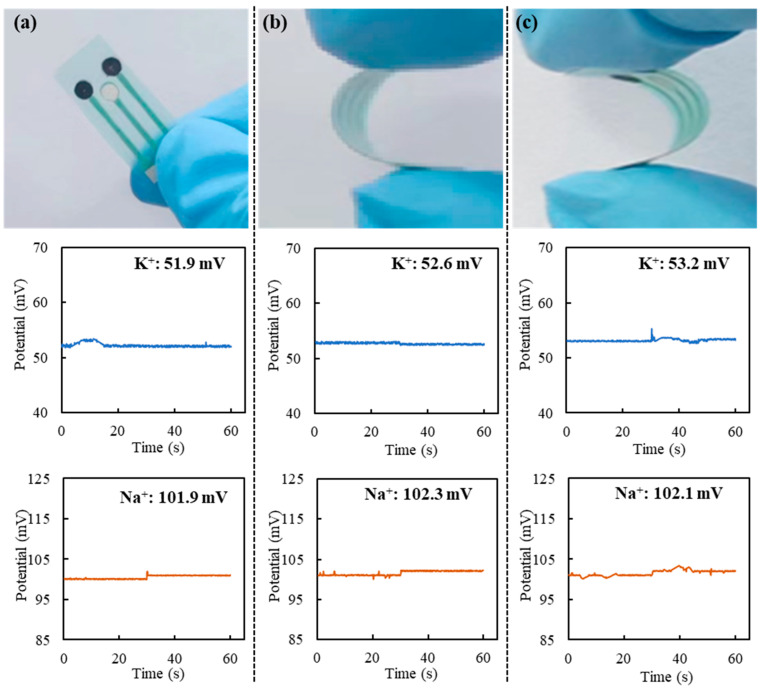
Potential response of K^+^ and Na^+^ ISEs of the sensor under mechanical deformations: (**a**) 0° bending; (**b**) 180° bending; (**c**) −180° bending while immersed in 2 mM KCl solution and 10 mM NaCl solution, respectively.

During the operation of the sensor, mechanical deformation occurs inevitably due to limb movement. Therefore, the resistance of the sensor to mechanical deformation was experimentally assessed. The test results are depicted in Figure 8. When subjected to mechanical deformations of 0° (Figure 8a), 180° (Figure 8b), and −180° (Figure 8c), the voltage variations of the K^+^ and Na^+^ ISEs relative to Ag/AgCl RE were found to be negligible, within a range of 2 mV. This indicates that the mechanical deformation of the sensor has a minimal impact on the K^+^ and Na^+^ concentration signals obtained from the sensors. Moreover, considering the sensor’s sensitivity of over 50 V decade^−1^, these changes are well within an acceptable range.

#### 3.2.5. Stability

**Figure 9:**
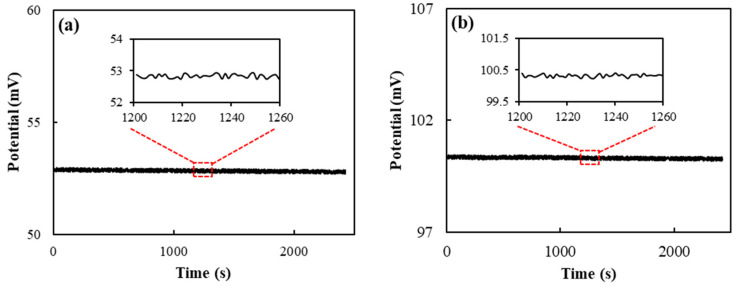
Recorded potential readings of the (**a**) K^+^ and (**b**) Na^+^ ISEs of the sensor when the sensor was immersed in a 2 mM KCl solution and a 10 mM NaCl solution for 40 min, respectively.

In order to assess the stability of the sensor during continuous monitoring of K^+^ and Na^+^ concentrations over an extended period of time (typically exceeding half an hour), a thorough evaluation was conducted. The results of the evaluation, depicted in Figure 9, reveal that once the K^+^ and Na^+^ signals obtained from the sensor reached a stable state, their respective voltage curves, namely K^+^ (Figure 9a) and Na^+^ (Figure 9b), exhibited a near-linear trend with minimal fluctuations within a range of 1 mV. These findings unequivocally demonstrate the exceptional stability of the sensor throughout the entire 40-minute detection period.

#### 3.2.6. Storage Stability

**Figure 10:**
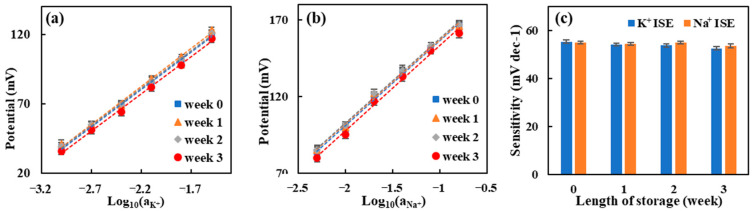
Storage stability (over three weeks) under ambient conditions: calibration curves of (**a**) K^+^ ISE and (**b**) Na^+^ ISE and (**c**) their sensitivities.

The storage stability of the screen-printed sensors was also investigated to assess their long-term viability. As depicted in Figure 10, the calibration curves for K^+^ and Na^+^ ions remained highly consistent over the initial two-week period, displaying a significant overlap. Towards the end of the test, a slight downward shift in the calibration curves was observed, indicating a minor variation. However, throughout the entire duration of the test, the sensitivity of the sensors showed no significant changes. These findings demonstrate the robust storage stability of the K^+^ and Na^+^ sensors fabricated using the screen-printing technique, further confirming their suitability for long-term storage and utilization in practical applications.

### 3.3. On-Body Trials

In order to validate the practical application effectiveness of the screen-printed sweat K^+^ and Na^+^ sensor, the sensor developed in this study was worn on a volunteer’s arms. The volunteer engaged in 40 min of daily running at the gym, and the variations in K^+^ and Na^+^ concentrations in the sweat were recorded to investigate their changing patterns. Additionally, changes in body weight were monitored and compared to variations in sweat ion concentrations.

**Figure 11:**
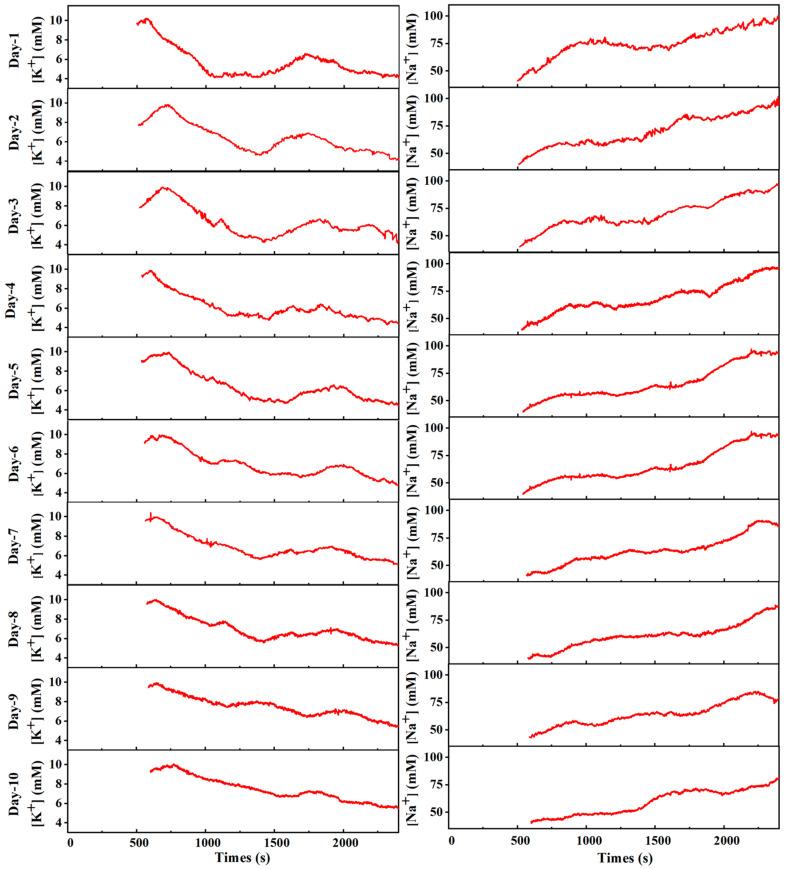
Concentration curves of K^+^ and Na^+^ ions in sweat secreted during a 40-minute daily treadmill running session with volunteers at the gym, as detected by the sensors developed in this study. The measurements were conducted continuously for 10 days, with each day consisting of a 40-minute running session.

Physical activity leads to the loss of water and electrolytes, including K^+^ and Na^+^, via sweating, which plays a vital role in regulating body temperature. A low concentration of K^+^ in sweat serves as an indicator of dehydration. Initially, sweat is nearly iso-osmotic to plasma, but as it travels through the ducts, sodium reabsorption occurs, resulting in a hypotonic state compared to plasma upon reaching the skin surface. The rate of sodium reabsorption depends on the sweat rate. Therefore, the Na^+^/K^+^ concentration can be correlated to an individual’s hydration status. Figure 11 illustrates the changes in K^+^ and Na^+^ concentrations in the volunteers’ sweat during their daily exercise sessions. It is evident that the concentrations of K^+^ and Na^+^ in sweat exhibited fluctuations throughout the exercise. Specifically, the K^+^ concentration in sweat gradually decreased with increasing exercise duration, whereas the Na^+^ concentration showed the opposite trend, increasing as the exercise duration prolonged. Moreover, the exercise monitoring data collected each day displayed consistent patterns, supporting the reliability of the findings, which align with the results reported in previous works [44,45,46].

The collected data encompassed a ten-day period, during which the volunteer engaged in regular exercise sessions. The recorded information included the changes in body weight, the starting response time of the sweat K^+^ and Na^+^ ISEs, the minimum K^+^ concentration, and the maximum Na^+^ concentration, as listed in Table 3. The analysis of these data revealed that over the ten-day period, the volunteer experienced a weight loss of 0.64 kg, indicating that short-term exercise alone does not yield rapid weight reduction without dietary modifications. However, as the duration of the exercise regimen increased, the observed changes in body weight before and after exercise diminished from 0.76 kg to 0.52 kg. This reduction indicated a decrease in sweat production. Additionally, the starting response time of the K^+^ and Na^+^ ISEs extended from 501 s to 597 s and from 498 s to 595 s, respectively, as the exercise days progressed. These results implied a delayed onset of sweat production with prolonged exercise duration. Furthermore, with an increasing number of exercise days, the minimum concentration of K^+^ in sweat during exercise exhibited an upward trend, while the maximum concentration of Na^+^ showed a decline. This observation suggests that exercise enhances the body’s resistance to dehydration.

In summary, systematic monitoring of the ion concentrations in the volunteer’s sweat during regular exercise sessions demonstrates that consistent physical activity can effectively reduce sweat volume per session and improve the body’s ability to withstand dehydration.

## 4. Conclusions

In this study, a wearable, flexible sensor capable of real-time monitoring of K^+^ and Na^+^ concentrations in human sweat was fabricated using screen printing technology. The sensor exhibits excellent performance, low production costs, and the ability for scalable mass production.

Initially, the electrical and electrochemical properties of the screen-printed substrate electrodes were thoroughly tested. The results confirmed their stable performance, meeting the requirements for sweat sensing applications. Subsequently, the sensitivity, linearity, repeatability, resistance to interferences, and mechanical deformation resistance of the fabricated K^+^ and Na^+^ ISEs were systematically evaluated. Additionally, the long-term stability during continuous detection and storage was assessed. The experimental outcomes demonstrated that the sensor, based on screen printing technology, exhibited outstanding detection performance for monitoring K^+^ and Na^+^ concentrations in human sweat. Furthermore, the developed sensor was utilized to periodically measure sweat during physical activity. Extensive data analysis revealed that regular and consistent exercise effectively enhances the body’s resistance to dehydration. These findings underscore the significance of wearable sweat sensors fabricated through screen printing technology in unraveling the intricate secrets concealed within human sweat and shedding light on its correlation with overall human health.

## Figures and Tables

**Table 1:** Conductivity performance of screen-printed substrate electrodes.

Electrodes	Types	Average Resistance (Ω)	Standard Deviation of Resistance (Ω)
K^+^ ISE	chip-to-chip (*n* = 5)	76.68	2.93
	batch-to-batch (*n* = 5)	76.56	4.33
Na^+^ ISE	chip-to-chip (*n* = 5)	76.48	2.11
	batch-to-batch (*n* = 5)	76.36	4.64
Ag/AgCl RE	chip-to-chip (*n* = 5)	1.22	0.08
	batch-to-batch (*n* = 5)	1.21	0.27

Note: K^+^ ISE, Na^+^ ISE, and the Ag/AgCl RE tested here are all the substrate electrodes, without any ISM or PVB coatings.

**Table 2:** Repeatability of calibration curves of the K^+^ ISE and Na^+^ ISE of the sensor.

	K^+^ ISE		Na^+^ ISE	
	Run-to-Run (*n* = 5)	Chip-to-Chip (*n* = 5)	Run-to-Run (*n* = 5)	Chip-to-Chip (*n* = 5)
average slope (mV/decade)	54.859	54.159	55.068	55.106
standard deviation of slope (mV/decade)	0.674	1.086	0.318	0.395
average base potential ^a^ (mV)	201.582	199.28	212	212.496
standard deviation of base potential (mV)	1.160	4.580	1.603	4.198

^a^ Intercept of the fitted calibration curve with the x = 0 axis.

**Table 3:** Daily weight changes in volunteers during 10 consecutive days of exercise, along with the variations in the starting response time of K^+^, Na^+^ ISEs, the minimum K^+^ ion concentration, and the maximum Na^+^ ion concentration recorded.

	Weight before Exercise (kg)	Weight after Exercise (kg)	s. r. ^a^ Time (s) of K^+^ ISE	min. con. ^b^ of K^+^ (mM)	s. r. Time (s) of Na^+^ ISE	max. con. ^c^ of Na^+^ (mM)
1st day	79.35	78.59	501	4.05	498	100.01
2nd day	79.41	78.75	512	4.11	508	99.55
3rd day	79.22	78.61	522	4.28	516	97.09
4th day	79.01	78.44	537	4.39	532	96.38
5th day	79.09	78.53	546	4.69	542	95.18
6th day	78.92	78.37	558	4.77	553	94.76
7th day	78.84	78.30	567	5.10	562	89.55
8th day	78.79	78.27	576	5.20	575	88.16
9th day	78.81	78.29	588	5.36	586	83.61
10th day	78.71	78.19	597	5.47	595	81.76

^a^ “s. r.” refers to starting response. ^b^ “min. con.” refers to minimum concentration. ^c^ “max. con.” refers to maximum concentration.

## Data Availability

The experimental data are available from the authors.

## Supplementary Materials

The following supporting information can be downloaded at: https://www.mdpi.com/article/10.3390/mi14081497/s1, Figure S1. Design and fabrication of the screen-printed substrate electrodes. (a) Graphic representation and dimensions of the Ag/AgCl layer; (b) graphic representation and dimensions of the carbon layer; (c) graphic representation and dimensions of the insulating layer; (d) physical appearance of the screen-printed substrate electrodes: Ⅰ-substrate electrode for K^+^ ISE, Ⅱ-substrate electrode for Na^+^ ISE, Ⅲ-substrate electrode for RE, Ⅳ-insulating layer. Figure S2. Signal conditioning circuit and the physical representation of the FPCB for the sensor. Figure S3. Open Circuit Potential (OCP) vs. time curves for the (a) K^+^ ISE and (b) Na^+^ ISE of the sensor, illustrating the temporal variation of OCP values from the initial unstable state to the gradual stabilization. The two electrodes were immersed individually in a 50 mL solution of 2 mM KCl and a 50 mL solution of 10 mM NaCl, respectively. Figure S4. Calibration curves with an extended concentration range (from 10^−7^ to 10^−1^ mol L^−1^) for (a) K^+^ ISE and (b) Na^+^ ISE of the sensor.
