# Supplementary material for: Screen-Printed Wearable Sweat Sensor for Cost-Effective Assessment of Human Hydration Status through Potassium and Sodium Ion Detection
Source: Micromachines (Basel). 2023 Jul 26;14(8):1497. doi: 10.3390/mi14081497 (PMC10456468; doi:10.3390/mi14081497)
Supplement: Supplementary file 1 [file micromachines-14-01497-s001.zip › micromachines-2499959-supplementary.pdf]

Screen-Printed Wearable Sweat Sensor for Cost-Effective Assessment of Human Hydration Status through Potassium and Sodium Ion Detection

Mingpeng Yang [1,2,*], Nan Sun [1], Xiaochen Lai [1,2], Yanjie Li [1], Xingqiang Zhao [1,2], Jiamin Wu [3] and Wangping Zhou [1,2,*]

1 School of Automation, Nanjing University of Information Science and Technology, 219 Ningliu Road,
Nanjing 210044, China; sunnan6047@163.com (N.S.); xchlai@nuist.edu.cn (X.L.); xx761563423@gmail.com.com (Y.L.); zxq8562@163.com (X.Z.)

2 Jiangsu Collaborative Innovation Centre on Atmospheric Environment and Equipment Technology,
Nanjing University of Information Science and Technology, 219 Ningliu Road, Nanjing 210044, China

3 Nanjing NARI Information and Communication Technology, Co., Ltd., 19 Chengxin Road,
Nanjing 211106, China; wjm19920727@163.com

* Correspondence: mpyang@mail.ustc.edu.cn (M.Y.); wpzhou@nuist.edu.cn (W.Z.);
Tel.: +86-17826058209 (M.Y.); +86-18951997253 (W.Z.)

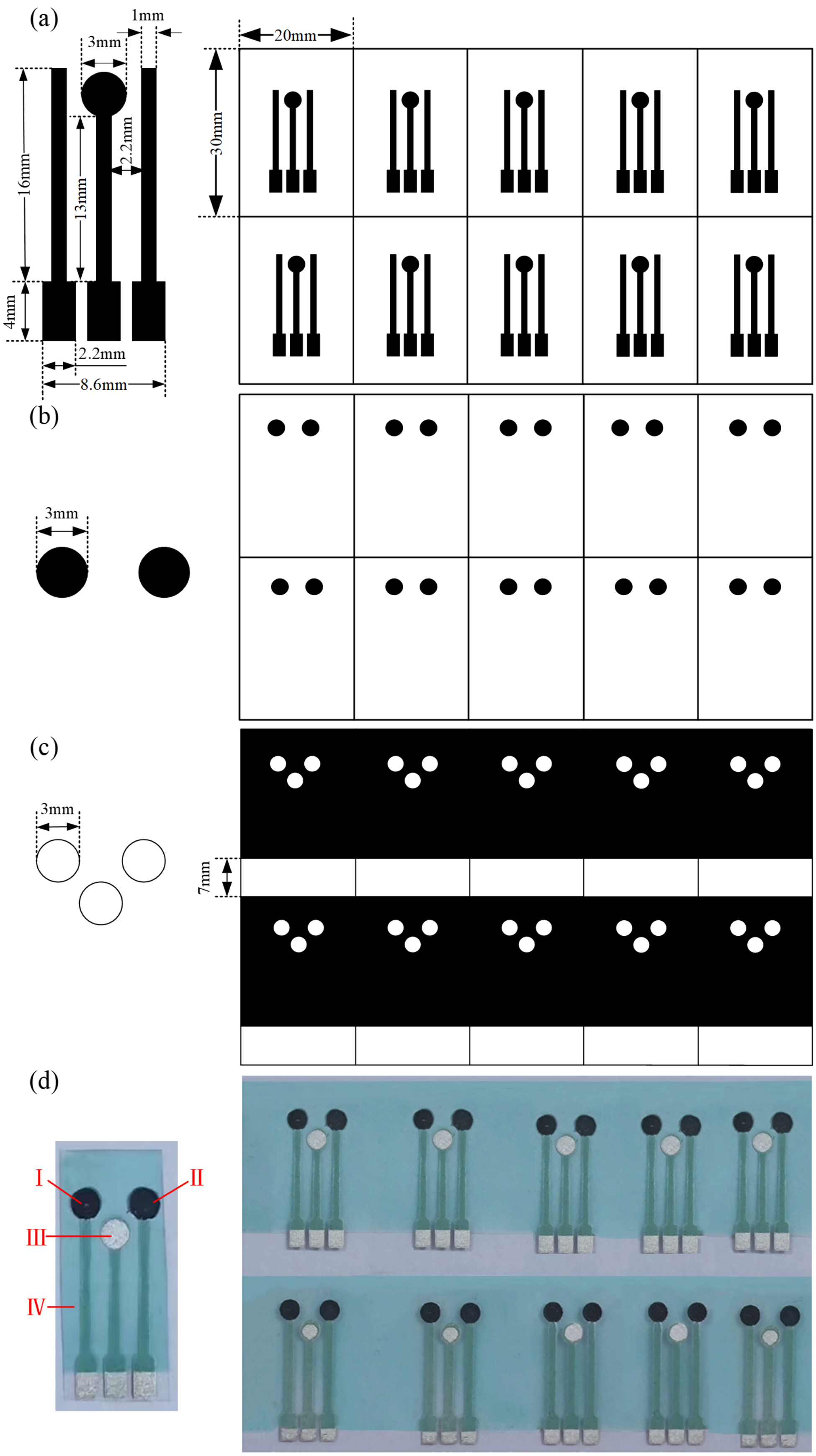

Figure S1. Design and fabrication of the screen-printed substrate electrodes. (a) Graphic representation and dimensions of the Ag/AgCl layer; (b) graphic representation and dimensions of the carbon layer; (c) graphic representation and dimensions of the insulating layer; (d) physical appearance of the screen-

printed substrate electrodes: I-substrate electrode for K^+ ISE, II-substrate electrode for Na^+ ISE, III-substrate electrode for RE, IV-insulating layer.

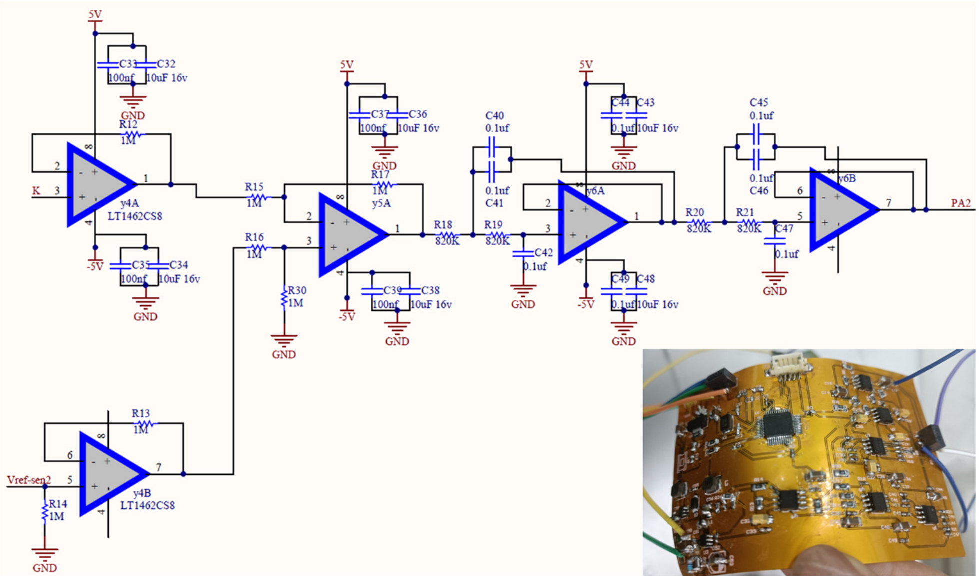

Figure S2. Signal conditioning circuit and the physical representation of the FPCB for the sensor.

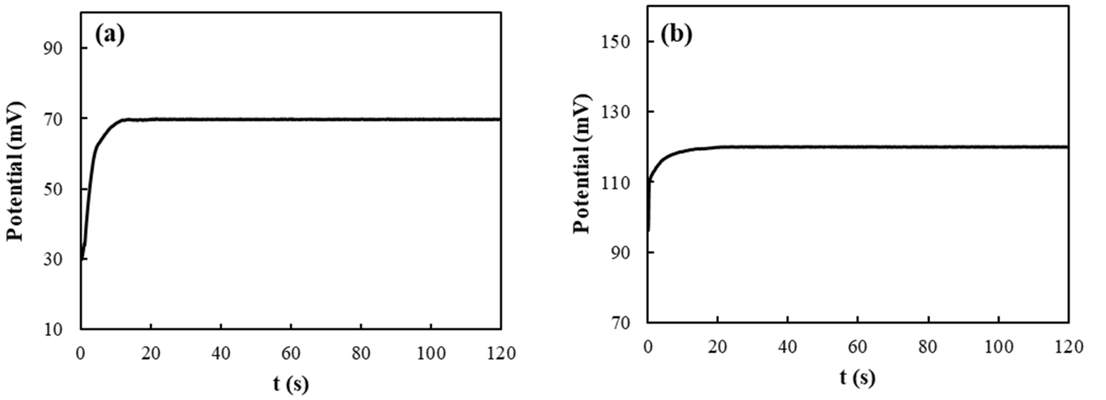

Figure S3. Open Circuit Potential (OCP) vs. time curves for the (a) K^+ ISE and (b) Na^+ ISE of the sensor, illustrating the temporal variation of OCP values from the initial unstable state to the gradual stabilization. The two electrodes were immersed individually in a 50 mL solution of 2 mM KCl and a 50 mL solution of 10 mM NaCl, respectively.

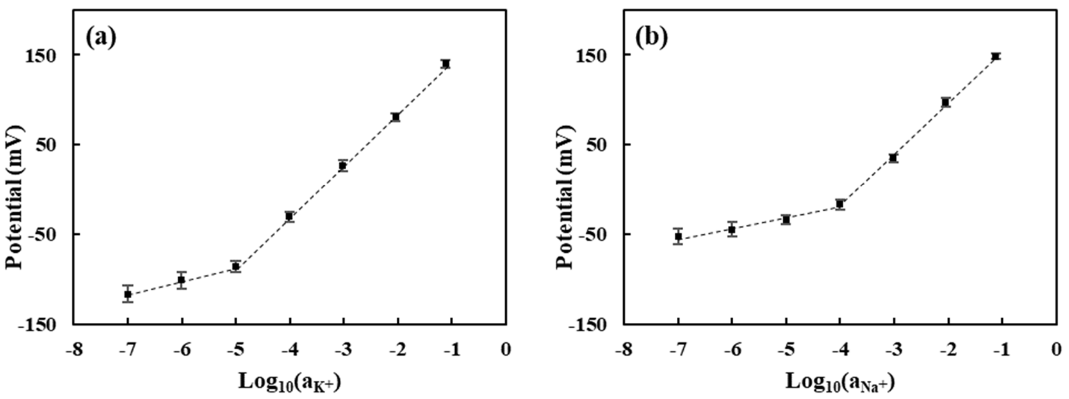

Figure S4. Calibration curves with an extended concentration range (from 10^{-7} to 10^{-1} mol L^{-1}) for (a) K^+ ISE and (b) Na^+ ISE of the sensor.
